# Hepatitis B testing practices at a tertiary care centre and their associated costs: A retrospective analysis

**DOI:** 10.1371/journal.pone.0219347

**Published:** 2019-07-08

**Authors:** Alexander Lawandi, Matthew P. Cheng, Todd C. Lee

**Affiliations:** 1 Division of Infectious Diseases, Department of Medicine, McGill University, Montréal, Québec, Canada; 2 Clinical Practice Assessment Unit, Department of Medicine, McGill University Health Centre, Montréal, Québec, Canada; Centers for Disease Control and Prevention, UNITED STATES

## Abstract

**Background:**

Hepatitis B is a viral infection requiring specific serologic testing to diagnose the stage of the disease. There are many tests which can be ordered in a variety of combinations. This study aimed to assess routine Hepatitis B screening practices in a tertiary care centre and determine the diagnostic and economic benefits of protocolized ordering.

**Methods:**

We evaluated all measurements of Hepatitis B total core antibodies, core IgM antibodies, surface antibodies and surface antigens performed at our institution between January 1, 2015 and December 31, 2015. We also recorded secondary testing (envelope antigens and antibodies, and viral DNA). Costs were estimated using provincial insurance reimbursement values. Using the subset of patients who received complete testing, we developed a reflexive screening protocol to minimize costs while simultaneously improving diagnostic utility.

**Results:**

30,335 hepatitis B tests were performed at an estimated total cost of $584,683. 53.9% of patients were screened with a single test. 29% of patients who received secondary testing had no evidence of exposure on primary testing. Using the protocol of initial testing of total core antibody and surface antibody with reflexive testing, we would save an estimated $181,632 (95% CI $154,201.90 –$208,910.50) per year while providing more complete information.

**Interpretation:**

Screening practices for Hepatitis B are frequently inadequate to diagnose and stage the infection and often included unnecessary testing. Protocolization of Hepatitis B testing could limit this practice while resulting in significantly lower costs.

## Introduction

Hepatitis B (HBV) is a common infection worldwide, with approximately 2 billion people having serologic evidence of past or current infection.[1] An estimated 30% of all individuals with cirrhosis can be attributed to chronic HBV infection.[2] Individuals infected with the virus will either spontaneously clear the virus or develop a chronic infection with a latency period that can span decades before the development of cirrhosis or hepatocellular carcinoma.[3]

While several professional societies have released guidelines on the management of both acute and chronic HBV infection,[4–6] appropriate management requires accurate diagnosis. The initial diagnosis of Hepatitis B infection hinges upon a combination of serologic titres (“primary testing”), including a measurement of the Hepatitis B core antibody and surface antibody and surface antigen. When infection is detected, subsequent testing (“secondary testing”) of viral DNA, envelope antigen and antibody titres, and liver enzymes is necessary in order to determine the stage of infection and the requirement for therapeutic intervention.[7] In most instances, “secondary” microbiological testing is only indicated in patients known to have a chronic Hepatitis B infection as indicated by the presence of circulating core antibody and surface antigen, although direct measurements of viral DNA can be clinically appropriate in highly selected cases.

Currently, many centres employ an open hepatitis B testing practice, in which tests are performed as ordered. Whether the test selection is appropriate, complete, or necessary is left to the discretion of the requesting physician. We sought to assess if protocolization of Hepatitis B testing could be diagnostically and economically advantageous by evaluating current practices at our institution which does not employ a standardized testing algorithm.

## Methods

All Hepatitis B total core antibody, surface antibody, surface antigen, envelope antigen, envelope antibodies and viral DNA quantification tests performed at the McGill University Health Centre (MUHC) (catchment area of 850,000) were included. The period of study was between January 1^st^, 2015 and December 31^st^, 2015. All patients 18 years of age and older who had testing done at the MUHC were included in the study, regardless of the location of their medical follow-up. All tests performed and resulted within the study time frame were extracted electronically from the health centre’s Laboratory Information System in an anonymized fashion, such that patient demographics were not available. Tests were grouped per the times at which they were ordered, such that tests ordered and performed on the same blood sample were treated as an individual set.

Measurements of Hepatitis B total core antibodies, core IgM antibodies, surface antibodies, surface antigens, envelope antigens, and envelope antibodies were measured via chemi-luminescence using the ARCHITECT i2000SR system (Abbott Diagnostics, Abbott Park, IL, USA). A protective surface antibody was defined as a test with a titre of 10IU/mL or greater. Hepatitis B viral DNA levels are measured quantitatively using the COBAS AmpliPrep/COBAS TaqMan automated system (Roche Molecular Systems, Inc., Branchburg, NJ, USA).

**Fig 1:**
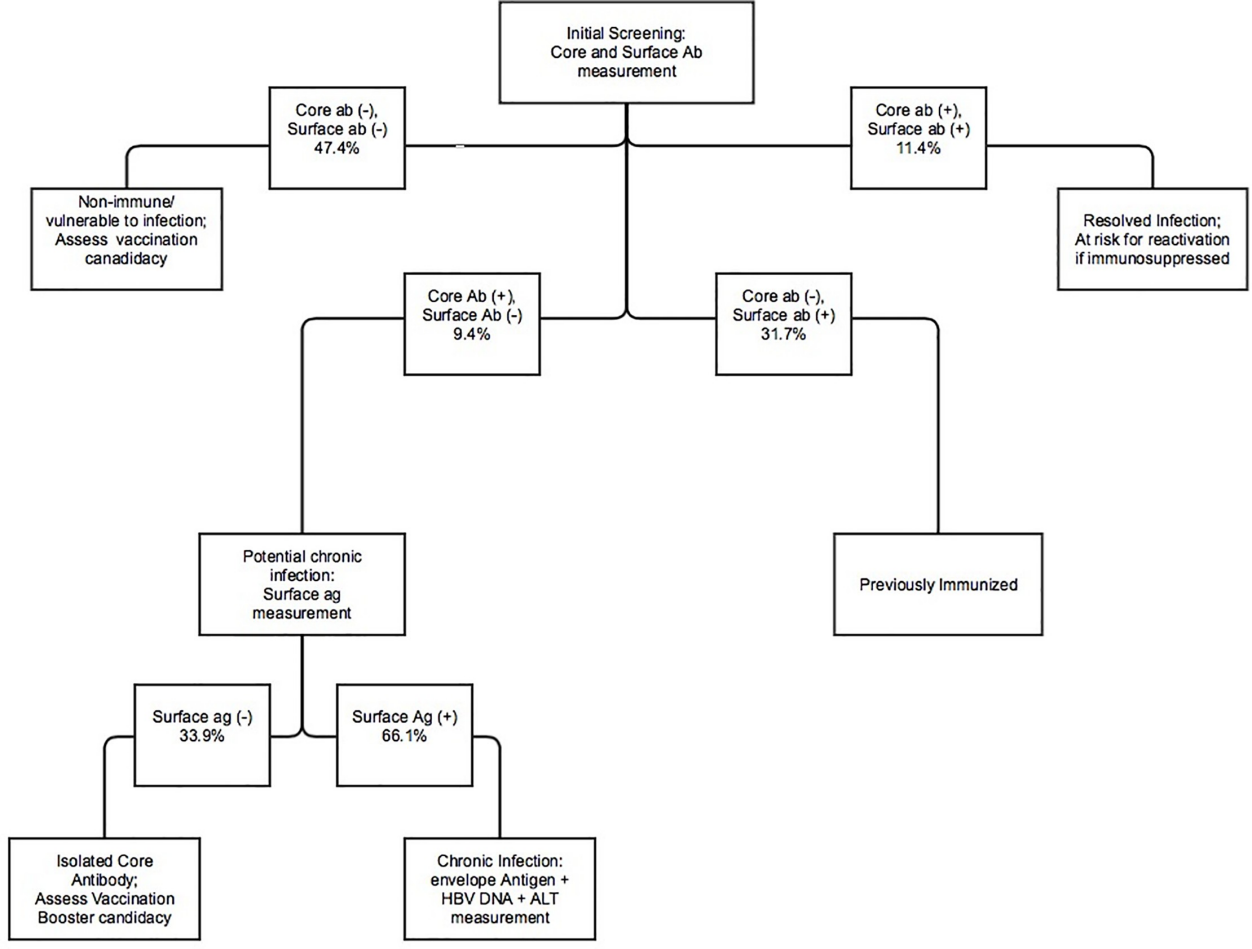
Proposed Hepatitis B Testing Algorithm; % represent the predicted number of patients with the particular testing results based upon the results of patients who completed the full primary screen; Testing to be performed reflexively according to algorithm.

Laboratory costs were estimated based on provincial insurance reimbursement values for Québec and are shown in Table 1. These costs account for the laboratory technologist’s time spent performing the assays as well as the cost of the assays themselves. We compared these actual per patient costs of all testing performed to an expected cost if an algorithmic approach was followed. In this theoretical testing algorithm, we propose that patients receive initial screening with Hepatitis B core IgG and surface antibody measurements with any further testing based on the combination of those results being automatically performed reflexively (Fig 1). Secondary testing is only performed when indicated and consists of measurement of surface antigen, followed by envelope antigen and viral DNA quantitation[8] as necessary.

**Table 1:** Estimated laboratory costs of testing per test type.

	cAb	sAb	sAg	envelope Ag	envelope Ab	HBV DNA
Estimated Cost (CAD$)	4.7	3.5	5.9	21	33	79

The goal of this algorithm was to ensure that initial screening was complete and that only patients who met specific criterion would receive secondary testing. The expected costs of using this algorithmic approach were calculated by taking the sum of the costs of tests performed at each stage in a protocolized approach multiplied by the probability of a patient proceeding to that stage of the algorithm and receiving those tests. These probabilities were inferred from the subgroup of our patients who had undergone complete serological testing. Confidence intervals around the probabilities were calculated using exact binomials and these were then factored into the calculations of the 95% confidence interval for the cost per patient.

Finally, we compared our proposed algorithm to one described by another group for the same local population more than decades prior.[9] Briefly, this algorithm tests Core IgG and does not pursue further testing if negative. If positive, Hepatitis B surface antigen is tested first, followed by surface antibody if the surface antigen is negative. This algorithm does not differentiate candidates who are immunized from those who could be. We compared our algorithm to theirs to estimate the additional cost to identify candidates for vaccination given the low cost and high efficacy of the immunization series.

The McGill University Health Centre Research Ethics Board approved this study. No external funding was utilized.

## Results

From January 1^st^ to December 31^st^, 2015, 26,962 patients received some form of primary Hepatitis B testing. The pattern of initial screening using the core antibody, surface antibody and surface antigen is reported in Table 2. Forty-five percent of testing was comprised of isolated surface antigen testing and the majority (78.9%) of testing was inadequate to properly assess hepatitis B status.

**Table 2:** Patterns of primary HBV testing ordered between January 1^st^, 2015 and December 31^st^, 2015.

	cAb alone	sAb alone	sAg alone	cAb + sAb	cAb + sAg	sAb + sAg	All 3	Total number of patients tested
N of patients receiving particular testing pattern (% of total tests)	403 (1.5)	2,624 (9.7)	11,507 (42.7)	344 (1.3)	1,205 (4.5)	4,907 (18.2)	5,972 (22.1)	26,962 (100)

**Fig 2:**
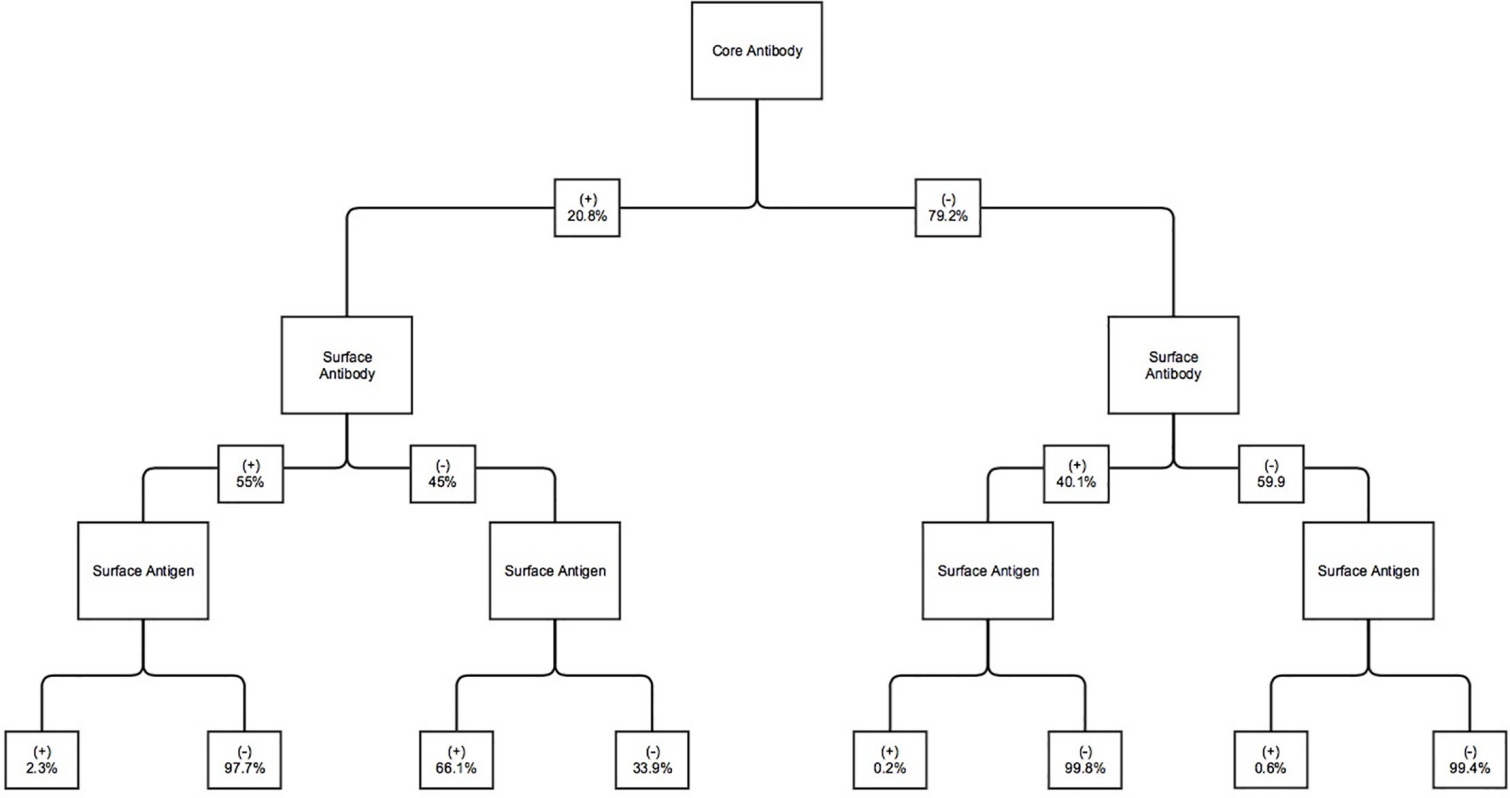
Breakdown of Primary Testing Results for Patients who received the full complement of testing (n = 5,972); Surface antibody is considered protective when it is measured as ≥10 IU/mL.

A complete set of primary tests was performed in 5,972 patients (22.1% of the total number of patients who underwent primary screening) and is shown in Fig 2. Within this group, the core antibody was negative in 4,728 patients (79.2%). Of these core antibody negative patients: 2,816 (59.6%) were negative for all 3 tests and therefore could be vaccinated; 1,892 (40.0%) had protective surface antibody titres (≥10 IU/mL) with negative surface antigen and therefore were immune from prior vaccination. 20 (0.4%) patients had positive surface antigen tests. Of these, 17 (85%) were found to be negative upon confirmatory testing with a surface antigen neutralization assay performed at the local reference centre. The remaining 3 (0.06% of total) did not have any further testing performed at our institution.

For the 1244 (20.8%) found to be core antibody positive: 190 (15.2%) had an isolated core antibody of unclear significance; 370 (29.7%) were positive for surface antigen, and 668 (53.7%) were positive for surface antibody. 16 (1.3%) were positive for all tests.

Core antibody IgM testing was performed in 758 patients and found to be positive in 6 patients (0.8%). Of these 6 positive results, 3 of had detectable total core IgG antibodies and surface antigens. Of the 758 core IgM measurements performed, 156 (20.6%) were performed without any other concomitant tests.

Secondary testing was performed in 4,778 patients during the study period (18.5%). The pattern of testing performed is shown in Table 3. Of these patients, only 1,386 (29.0%) had core antibody testing. 210 (15.2%) of these patients received secondary testing despite being core antibody negative, most commonly with measurements of viral DNA (44.8%). The largest proportion of patients receiving secondary testing (71%) did not have core antibody testing performed during the study period.

**Table 3:** Patterns of secondary testing performed, according to core antibody status.

		eAg test alone	eAb test alone	DNA alone	eAg + eAb	eAg + DNA	eAb + DNA	All 3	Total
N (% of total tests per category)	cAb (+)	29 (2.5)	8 (0.7)	672 (57.1)	139 (11.8)	97 (8.2)	7 (0.6)	223 (20)	1,176
	cAb (-)	48 (22.8)	30 (14.3)	94 (44.8)	25 (11.9)	2 (0.9)	0 (0)	11 (5.2)	210
									1,386

The total cost of testing in calendar year 2015 was CAD$584682.60 (CAD$21.68 per patient). Following the proposed protocol (Fig 1) and using the proportions of patients in each group as determined by our sample of patients who received complete primary investigation, we estimate the cost per patient would have been CAD$14.95 (95% CI CAD$13.94-$15.97), representing a savings of more than 30% (CAD$181632.30 per year, 95% CI CAD$154,201.90-$208,910.50). This approach would provide complete information regarding immunity as compared to the alternative algorithm [9] which was slightly less expensive per patient (CAD$12.89 per patient, 95% CI CAD$11.99-$13.78).

## Discussion

We have demonstrated that in the absence of a testing protocol implemented at the level of the clinical laboratory, patients may receive inadequate primary screening for Hepatitis B infection as well as inappropriate secondary testing. The plethora of possible tests for the assessment of Hepatitis B infection can challenge clinicians unfamiliar with the intricacies of laboratory testing and this can lead to wasted laboratory resources, delays in diagnosis, and missed opportunities for vaccination and treatment. In our study, 54% of patients were screened with a single test. While there exist recommendations to initiate screening with measurement of surface antigen first[10], particularly for people at elevated risk for Hepatitis B infection, there are limitations to this method. Most notably, a negative test cannot distinguish between unexposed, immunized, and those previously infected who have seroconverted. Moreover, while a nuanced approach to testing based on risk is logical, it also adds complexity which may further increase variation in physician practice. Therefore, we believe our protocolized approach offers advantages over an approach in which surface antigen is performed as the first test with a very low rate of potential misclassification. We do concede that in the setting of acute infection, in which the surface antigen may be the only positive marker, our proposed algorithm may fail to accurately identify these patients. However, in settings of low endemic transmission such as Canada, this will be a minority of the cases. Physicians who are concerned about acute infection should always have the ability to contact the clinical laboratory and specifically request the inclusion of the surface antigen measurement initially alongside the core and surface antibodies. Similarly, in the setting of the HIV infected population, a study has demonstrated that there can be occult HBV viremia as evidenced by detectable viral DNA in blood while the surface antigen is negative [11]. Consequently, for specific high-risk patients, clinicians should be aware of this possibility and the need to contact the laboratory and request measurement of the viral DNA outside of the protocol.

As with surface antigen, the measurement of a surface antibody alone can not distinguish between resolved natural infection and immunization. Similarly, the measurement of core antibody alone cannot distinguish between active and resolved infection. Therefore, a combination of these tests is required to correctly infer the patient’s status. This is of particular importance for patients undergoing certain chemotherapies, as identification of patients with positive core antibodies can require careful follow up as they may be at elevated risk for Hepatitis B reactivation.[12] If only the surface antigen and antibody are used in the initial assessment, previously exposed patients can be missed with potential morbidity if subsequently immunosuppressed.

We also noted that several patients received primary screening with core IgM testing alone. This is particularly worrisome as a negative IgM does not exclude the possibility of a chronic Hepatitis B infection and could lead to missed diagnoses.

In terms of secondary testing, we found that an important percentage of patients received testing that was not medically indicated. Except in rare circumstances, there are few indications for the testing of Hepatitis B viral DNA when a patient’s core antibody is negative, including in individuals with inherited or acquired antibody deficiencies. Similarly, envelope antigen and antibody titres should also only be measured when patients are known for positive core and surface antigen. In the setting of testing as ordered, non-informative serologic results appear to be common. Our proposed algorithm would reflexively lead to measurement of viral DNA and envelope antigen in patients found to be core antibody positive and surface antigen positive but would not include the envelope antibody measurement. This omission is intentional as the most recent recommendations suggest treatment strategies according to the ALT, viral DNA and envelope antigen alone. [13] While envelope antibody is important in monitoring for seroconversion, the initial screening and treatment decisions are not based upon it and therefore can be excluded from an initial screening algorithm. Similar to the described situation for acute hepatitis B infection, clinical laboratories implementing the proposed algorithm would need to allow for envelope antibody measurements to be performed upon specific request.

A striking finding of this study was the potential cost savings that would accrue if a protocolized approach to Hepatitis B screening was implemented. Our results suggest that costs at our centre could be reduced by over 30% using this approach. If one were to consider the potential benefits of identification of patients at risk for de novo Hepatitis B infection or reactivation in the face of immunosuppression, these results likely under-represent the potential savings. While our protocolized approach appears to be more costly than the comparator algorithm, it will provide more complete information. Given that hepatitis B is vaccine preventable and both community and nosocomial transmission still occur in Canada, we favor an approach that determines immunity and provides the opportunity for vaccination in patients for whom their physicians have already decided to test for Hepatitis B.

Our study has several limitations. Firstly, the data was collected retrospectively and at the level of the laboratory. The clinical rationale guiding the testing was not determined. However, we feel that the results demonstrating unusual combinations of tests ordered, as well as the amount of secondary testing performed on core antibody negative patients is suggestive of a poor understanding of the serologic diagnosis of Hepatitis B. Secondly, an important percentage of patients underwent repeat testing during the study period. The nature of these repeats was not explored but could suggest, in some cases, a staggered approach to testing was being implemented to account for incomplete initial primary screening. However, numerous patients with documented core antibody positivity underwent repeated core antibody measurements within the study period, reaffirming either an incomplete understanding of the meaning of the result or insufficient review of previous testing results prior to further testing. While not investigated within this study, further protocolization to prevent repeated core antibody measurements in patients known for a positive result would likely add to cost savings. A third important limitation of our study is that our results may not be generalizable to populations with different prevalence of natural infection and immunization; to centres who use serologic tests with less sensitivity; or to centres where the relative costs between the tests are substantially different. This study relied upon data extracted from the Laboratory Information System and patient level data such as demographics could not be determined. As a result, the ability to extrapolate the results to specific populations is reduced. Finally, it is important to note that the algorithm we propose may miss early hepatitis B infection prior to the production of core antibody or infection in patients with antibody deficiencies. In patients where this is a significant concern, additional testing for surface antigen would be warranted and any laboratory protocol must allow for such exceptions to be communicated when testing is ordered and deviations from the algorithm to be processed upon specific request.

In conclusion, we have demonstrated that the complex nature of the serologic assessment for Hepatitis B infection leads to wide variations in practice coupled with inadequate and inefficient testing. Implementation of a testing algorithm as we have proposed would substantially improve both the interpretability and value of our testing results. Future studies should investigate assess the benefits of protocolization of testing in terms of total number of tests performed as well as in the time to accurate diagnosis of Hepatitis B status.
